# Clostridium difficile Modulates the Gut Microbiota by Inducing the Production of Indole, an Interkingdom Signaling and Antimicrobial Molecule

**DOI:** 10.1128/mSystems.00346-18

**Published:** 2019-03-19

**Authors:** Charles Darkoh, Kimberly Plants-Paris, Dayna Bishoff, Herbert L. DuPont

**Affiliations:** a University of Texas Health Science Center, School of Public Health, Department of Epidemiology, Human Genetics, and Environmental Sciences, Center For Infectious Diseases, Houston, Texas, USA; b Microbiology and Infectious Diseases Program, UTHealth Graduate School of Biomedical Sciences, University of Texas MD Anderson Cancer Center, Houston, Texas, USA; University of California, San Francisco

**Keywords:** *C. difficile*, indole, *C. difficile* infection, indole production, gut microbiota, tryptophan biosynthesis, tryptophan metabolism

## Abstract

*Clostridium* (*Clostridioides*) *difficile* infection (CDI) is associated with dysbiosis. C. difficile has a characteristic propensity to persist and recur 1 to 4 weeks after treatment, but the mechanism is unknown. We hypothesized that C. difficile may persist by manipulating the intestinal microenvironment, thereby hampering gut microbiota reconstitution following antibiotic-mediated dysbiosis. By screening stools from CDI patients for unique markers, a metabolite identified to be indole by mass spectrometry and Fourier transform infrared spectroscopy was identified. The average fecal indole concentration detected in CDI patients (*n* = 216; mean, 1,684.0 ± 84.4 µM) was significantly higher than in stools of patients with non-C. difficile diarrhea (*n* = 204; mean, 762.8 ± 53.8 µM). Certain intestinal bacteria, but not C. difficile, produce indole, a potent antimicrobial antioxidant. Remarkably, C. difficile induced other indole-producing gut microbes to produce increasing amounts of indole. Furthermore, a C. difficile accessory gene regulator 1 quorum sensing system mutant cannot induce indole, but complementation of the mutant strain with the wild-type gene restored its ability to induce indole production. Indole tolerance assays indicated that the amount of indole required to inhibit growth of most gut-protective bacteria was within the range detected in the CDI stools. We think that a high indole level limits the growth of beneficial indole-sensitive bacteria in the colon and alters colonization resistance and this might allow C. difficile to proliferate and persist. Together, these results reveal a unique mechanism of C. difficile persistence and provide insight into complex interactions and chemical warfare among the gut microbiota.

**IMPORTANCE** Clostridium difficile infection is the leading cause of hospital-acquired and antibiotic-associated diarrhea worldwide. C. difficile flourishes in the colon after the diversity of the beneficial and protective gut microbiota have been altered by antibiotic therapy. C. difficile tends to persist, as does dysbiosis, encouraging recurrence a few days to weeks after treatment, and this further complicates treatment options. Here, we show that C. difficile might persist by manipulating the indigenous microbiota to produce indole, a bioactive molecule that inhibits the growth and reconstitution of the protective gut microbiota during infection. This discovery may explain a unique strategy C. difficile uses to control other bacteria in the colon and provide insight into the complex interactions and chemical warfare among the gut microbiota.

## Abstract (precis)

Clostridium difficile infection is the leading cause of hospital-acquired and antibiotic-associated diarrhea worldwide. C. difficile flourishes in the colon after the diversity of the beneficial and protective gut microbiota have been altered by antibiotic therapy. C. difficile tends to persist, as does dysbiosis, encouraging recurrence a few days to weeks after treatment, and this further complicates treatment options. Here, we show that C. difficile might persist by manipulating the indigenous microbiota to produce indole, a bioactive molecule that inhibits the growth and reconstitution of the protective gut microbiota during infection. This discovery may explain a unique strategy C. difficile uses to control other bacteria in the colon and provide insight into the complex interactions and chemical warfare among the gut microbiota.

## INTRODUCTION

The commensal gut microbiota promote human health by defending the gastrointestinal tract against colonization by pathogenic bacteria (1, 2). Some of the factors that contribute to the protective role of the gut microbiota include competition for nutrients (3), adhesion sites (4, 5), production of antimicrobials to ward off pathogens (6, 7), and modulation of the host’s immune defense mechanisms against pathogens (8). Consequently, disruption of the abundance and diversity of the gut microbiota leads to increased susceptibility and colonization of certain pathogens such as Clostridium difficile (8, 9).

C. difficile is the leading cause of hospital- and antibiotic-associated diarrhea worldwide. It is at the top of the list of pathogens designated an urgent public health threat by the U.S. Centers for Disease Control and Prevention. C. difficile is resistant to multiple antibiotics and overpopulates the colon after the protective gut microbiota have been altered by antibiotic therapy. Following colonization, it produces toxins A and B that cause severe intestinal inflammation, diarrhea, and pseudomembranous colitis. The accessory gene regulator 1 (Agr1) quorum signaling system regulates the production of these toxins (10, 11). This is mediated by the release of a cyclic autoinducing peptide (TI signal) by the growing C. difficile cells, which accumulates and activates toxin production upon reaching a threshold concentration. Whether the TI signal interacts with the other gut microbiota was unknown until now. Moreover, C. difficile is unique among the enteric pathogens because of its characteristic propensity to persist in the gut and recur 1 to 4 weeks following treatment of the primary infection. An estimated 20% to 30% of patients with primary C. difficile infection (CDI) experience recurrence within 2 weeks after completion of therapy, and these patients exhibit persistent dysbiosis (12, 13). It is unclear why intestinal dysbiosis persists after CDI, which also promotes recurrent infections. While spore formation is important in CDI recurrence, we hypothesized that C. difficile may persist by direct manipulation of the intestinal microenvironment to hamper reconstitution of the gut microbiota following antibiotic-associated dysbiosis.

Indole is widely distributed in nature and a major component of various essential compounds known for their medicinal properties. Analogs of indole are also utilized in various industrial applications such as dyes, plastics, flavor enhancers, vitamin supplements, agriculture, over-the-counter drugs, and perfumery. Indole is mainly produced from tryptophan by certain gut microbiota using tryptophanase, an enzyme that hydrolyses tryptophan into indole, pyruvate, and ammonia (14). Approximately 85 known genera of Gram-positive and Gram-negative bacteria of the phyla *Bacteroidetes*, *Firmicutes*, *Proteobacteria*, and *Actinobacteria*, excluding C. difficile, produce tryptophanase (14). Depending on the diet, fecal indole concentrations in healthy adults range from 0.30 to 6.64 mM (15). Indole can also be metabolized by certain bacteria to tryptamine or indole-3-acetic acid; the latter is further converted to 3-methyl indole (16). Hepatocytes metabolize some indole analogs into indoxyl-3-sulfate. Two other indole metabolites, indole-3-aldehyde and indole-3-propionic acid, are produced by the gut commensals *Lactobacillus* spp. (17) and Clostridium sporogenes (18), respectively.

In the bacterial kingdom, indole plays a signaling role and controls diverse physiological processes, such as antimicrobial response (19), biofilm formation (20, 21), motility (20), persister cell formation (22), plasmid stability (23), and virulence (24). Indole also regulates interspecies communication and host cell invasion by non-indole-producing microbes such as Pseudomonas aeruginosa, Salmonella enterica, and Candida albicans (25–28). In the mammalian host, indole and other metabolites derived from tryptophan modulate inflammation in the gastrointestinal tract (29–31) and support epithelial tight junction permeability (29). Indole also acts on host tissues by enhancing barrier functions, maintaining intestinal homeostasis, increasing mucus production, decreasing the production of inflammatory markers, increasing the production of anti-inflammatory molecules, and increasing the expression of a wide variety of genes associated with the immune system (20, 30, 32, 33). Hence, indole and its derivatives play important roles in the pathophysiology of both eukaryotic and prokaryotic organisms.

In Escherichia coli, the expression of tryptophanase is regulated by a 3,144-bp *tna* operon comprising three genes, *tnaL*, *tnaA*, and *tnaB*. *tnaA* and *tnaB* are the major structural genes that encode tryptophanase and tryptophan permease, respectively (34–36). Upstream of *tnaA* is *tnaL*, which encodes a 25-residue leader peptide. The *tna* operon is under catabolic repression and regulates the use of tryptophan as carbon and nitrogen sources (37–39). Several species of both Gram-negative and Gram-positive bacteria have homologues of the *tna* operon genes and are known to produce indole (14). Here, we demonstrate that C. difficile (a non-indole producer) induces high indole production among the indole-producing gastrointestinal bacteria, which may have significant consequences on the abundance and diversity of the bacterial communities in the colon.

## RESULTS

**FIG 1:**
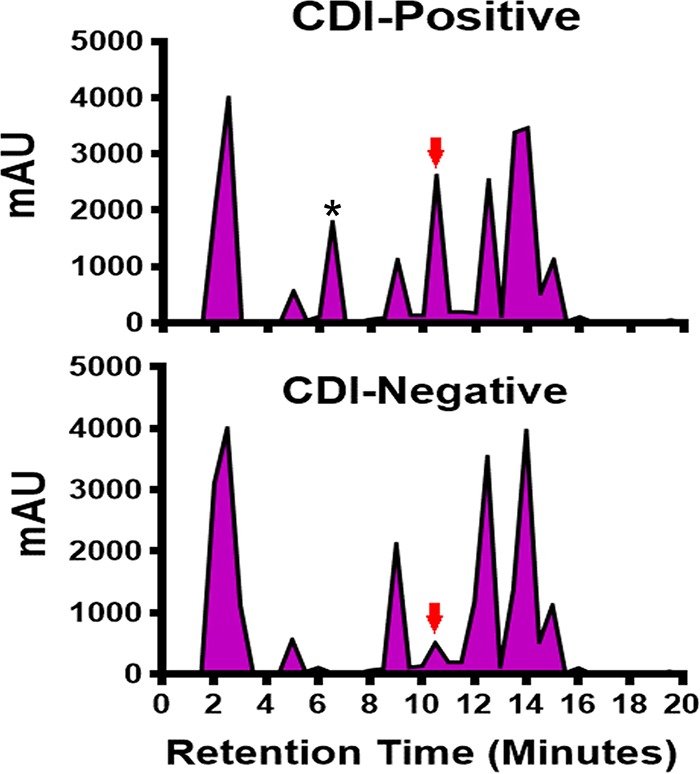
Comparative analysis of metabolites present in CDI-positive and CDI-negative stools by preparative high-performance liquid chromatography using a C_18_ column. Stools (1 g each) from 20 diarrhea patients infected with C. difficile and 20 diarrhea patients without C. difficile were pooled, suspended in PBS, and acetate precipitated. The precipitated material was resuspended in PBS and filtered through a centrifugal filter with a 3-kDa-cutoff membrane, and the filtrate was concentrated in a SpeedVac (Thermo Fisher Scientific, Waltham, MA). The concentrated filtrate was injected into a preparative Econosil C_18_ column (250 mm by 10 mm; Alltech) and purified using a Shimadzu Prominence HPLC system (Shimadzu Scientific Instruments, Columbia, MD). Each purification run was performed by injecting 1 ml of the sample and washing with buffer A (97.5% [vol/vol] acetic acid-H_2_O; pH 3.8), followed by gradient elution with buffer B (80%:20% [vol/vol] acetonitrile/H_2_O). The red arrow indicates the unique indole peak. *, peak contained the autoinducing quorum signaling peptide (TI signal) associated with C. difficile toxin regulation. A representative chromatogram from multiple runs is shown. mAU, milli-arbitrary units.

To identify unique C. difficile-associated metabolites, stool samples from diarrhea patients confirmed to be CDI positive and CDI negative were analyzed on a C_18_ column. A unique peak was observed in the CDI-positive stools at elution times between 10 to 11 min whose area under the curve was consistently higher than that of the CDI-negative samples (Fig. 1). The fraction associated with this peak was further purified, and analysis of the purified material by mass spectrometry revealed a predominant compound with a molecular weight of 116.5 Da, which was consistent with indole. Further analysis by gas chromatography-mass spectrometry and Fourier transform infrared spectroscopy demonstrated that the spectra from the purified material were identical to those from indole (see Fig. S1 in the supplemental material).

10.1128/mSystems.00346-18.1 FIG S1 Confirmation of the purified patient stool-derived material as indole by gas chromatography-mass spectrometry (A) and Fourier transform infrared spectroscopy analysis (B). Stools (1 g each) from 20 diarrhea patients infected with C. difficile and 20 diarrhea patients without C. difficile were pooled, suspended in PBS, and acetate precipitated. The precipitated material was resuspended in PBS and filtered through a centrifugal filter with a 3-kDa-cutoff membrane, and the filtrate was concentrated in a SpeedVac (Thermo Fisher Scientific, Waltham, MA). The concentrated filtrate was injected into a C_18_ column and purified using a Shimadzu Prominence HPLC system (Shimadzu Scientific Instruments, Columbia, MD). Each purification run was performed by injecting 1 ml of the sample and washing with buffer A (97.5% [vol/vol] acetic acid-H_2_O; pH 3.8), followed by gradient elution with buffer B (80%:20% [vol/vol] acetonitrile/H_2_O). A unique peak that was significantly associated with the CDI-positive stools was further purified and analyzed by gas chromatography-mass spectrometry (A) and Fourier transform infrared spectroscopy (B). Download FIG S1, TIF file, 0.1 MB. Copyright © 2019 Darkoh et al. 2019 Darkoh et al. This content is distributed under the terms of the Creative Commons Attribution 4.0 International license.

**FIG 2:**
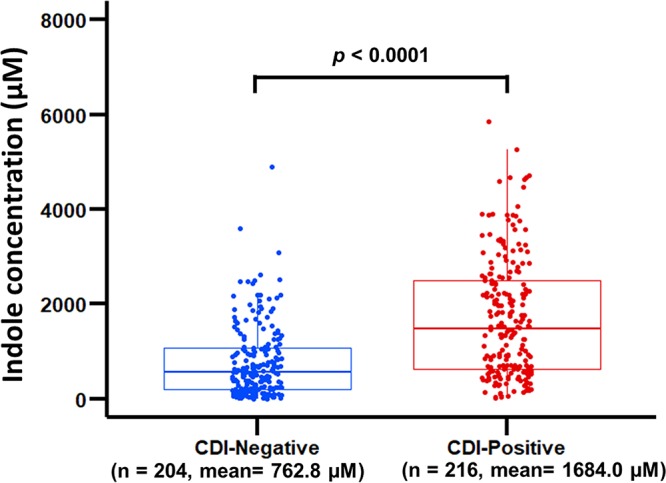
Indole level in stools of CDI-positive patients is significantly higher than that of CDI-negative patients. Stool samples (250 mg) from each patient were suspended in 750 µl of 70% ethanol, incubated in a water bath at 70°C for 10 min, and centrifuged at 14,000 × *g* for 20 min at 40°C. Indole concentration in the supernatant was determined in triplicates using the hydroxylamine indole assay (15). CDI-positive patients (mean, 1,684.0 ± 84.4 µM; median, 1,476.0 µM; range, 14.4 to 5,847.0 µM); CDI-negative patients (mean, 762.8 ± 53.8 µM; median, 551.6 µM; range, 8.1 to 4894 µM). Mann-Whitney U test indicated a significant difference (*P < *0.0001) in indole levels between the CDI-positive and CDI-negative diarrhea stools. Each data point represents the average from three independent replicate experiments.

Based on these initial results, we questioned whether indole levels in diarrhea stools of CDI patients would be different from stools of diarrhea patients without CDI. To investigate this question, indole concentrations in stools of 216 CDI-positive and 204 CDI-negative diarrhea patients were determined. The mean and median indole concentrations in stools of the CDI-negative patients were 762.8 ± 53.8 µM and 551.6 µM, respectively, and ranged from 8.1 to 4,894 µM (Fig. 2). Surprisingly, indole levels in stools from the CDI patients were higher with a mean concentration of 1,684.0 ± 84.4 µM, a median of 1,476.0 µM, and ranged from 14.4 to 5,847.0 µM. A Mann-Whitney U test indicated that the overall difference in stool indole concentration between the CDI-positive and CDI-negative patients was statistically significant (*P < *0.0001). The mean and median fecal indole concentrations from 53 healthy adults have been reported to be 2.59 mM and 2.73 mM, respectively, with a range of 0.30 mM to 6.64 mM (15). These results demonstrated that indole levels in stools of CDI patients were higher than in stools of CDI-negative diarrhea patients.

### C. difficile induces indole production.

**FIG 3:**
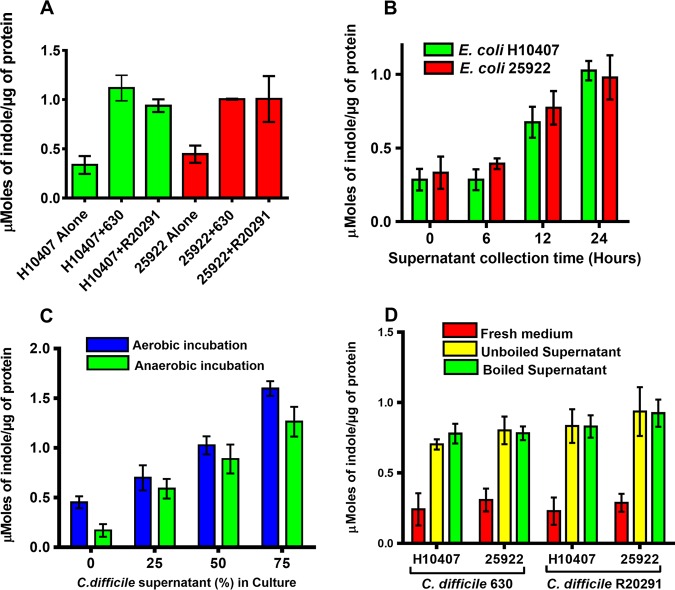
C. difficile induces indole production in enterotoxigenic E. coli. (A) E. coli strains H10407 and 25922 were cocultured anaerobically for 24 h at 37°C with either C. difficile R20291 or 630 strain. Mann-Whitney U test showed the amount of indole produced was significant between E. coli cultured alone versus E. coli cocultured with C. difficile (*P < *0.0001). The amount of indole induced was not significant between the different C. difficile strains (*P = *0.2756). (B) E. coli strains were cultured aerobically for 5 h in brain heart infusion (BHI) medium containing 50% C. difficile R20291 culture supernatant collected at different growth periods, namely, 6 h (mid-log phase), 12 h (early stationary phase), and 24 h (late stationary phase), and indole levels were tested. One-way ANOVA indicated significant differences in amounts of indole induced by the supernatants collected from different growth stages of C. difficile (*P < *0.0001). (C) E. coli strains were cultured either aerobically or anaerobically for 5 h at 37°C in BHI medium containing different amounts of the C. difficile R20291 supernatant and indole levels were tested. One-way ANOVA indicated significant differences in the amounts of indole induced by the different supernatant amounts (*P < *0.0001). (D) E. coli strains were cultured aerobically for 5 h at 37°C in BHI medium containing either boiled or unboiled supernatants from C. difficile strains R20291 or 630. Mann-Whitney U test showed no significant difference (*P = *0.8515) in the amounts of indole induced between boiled and unboiled supernatants. Indole concentration was determined in triplicates using the hydroxylamine indole assay (15). The error bars represent the standard deviations from three replicate experiments.

The high indole level observed in the stools of CDI patients was unusual because none of the sequenced genomes of C. difficile strains encode a homolog of the tryptophanase gene required to make indole. As a result, C. difficile strains cannot produce indole. We rationalized that perhaps the presence of C. difficile might be stimulating indole production in other gut bacteria that encode the tryptophanase gene. To examine this, C. difficile cells or their culture supernatants were cocultured with known indole-producing bacteria. Remarkably, E. coli strains H10407 and 25922 cocultured with either C. difficile strain R20291 or 630 produced significantly (*P < *0.0001) more indole than when cultured alone (Fig. 3A). Also, the amount of indole induced by C. difficile strain 630 was not significantly different from that induced by the R20291 strain (*P *= 0.2756). These results suggested that C. difficile induces indole production in a non-strain-specific manner. To investigate whether C. difficile culture supernatant fluids can also induce indole, E. coli strains were incubated with culture supernatants collected from different growth stages of the R20291 strain. E. coli H10407 and 25922 strains incubated aerobically with C. difficile stationary-phase supernatants (12 h and 24 h) produced significantly (*P* < 0.0001) more indole than when incubated without C. difficile supernatant (Fig. 3B). On the other hand, the amount of indole produced when the E. coli cells were exposed to the log-phase C. difficile supernatant was not different from the amount produced when cultured alone, indicating that the indole-inducing factor either accumulates during growth or is produced during stationary phase. Furthermore, the E. coli strains produced indole in a dose-dependent manner under both aerobic and anaerobic conditions in the presence of the C. difficile culture supernatant (Fig. 3C). In addition, both boiled and unboiled C. difficile supernatant fluids induced indole production in the E. coli strains (Fig. 3D), suggesting that the inducing factor is not significantly (*P = *0.8515) affected by high temperature.

### C. difficile induces *tnaA* expression.

**FIG 4:**
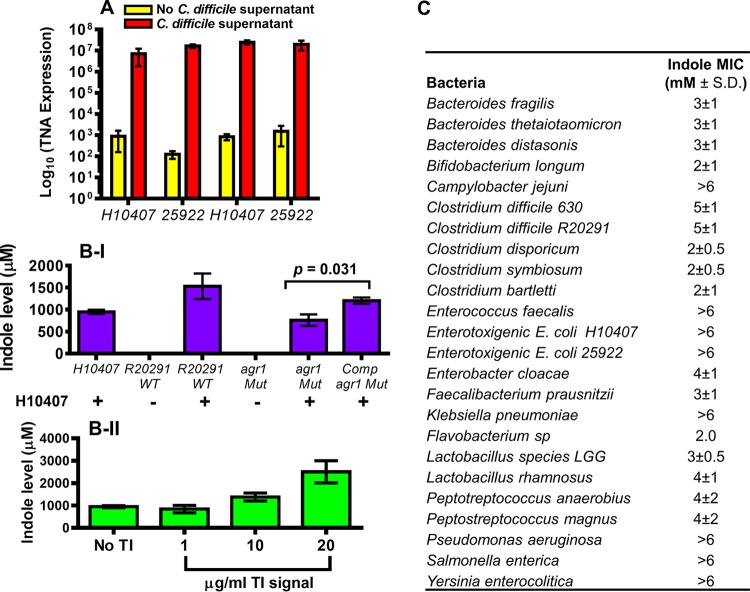
C. difficile induces *tnaA* overexpression in enterotoxigenic E. coli. (A) E. coli strains H10407 and 25922 were incubated anaerobically for 6 h at 37°C in fresh BHI medium containing 25% 0.2-µm-filtered cell-free C. difficile R20291 supernatant and 5 mM l-tryptophan. Total RNA was isolated using an RNeasy kit (Qiagen), and this was followed by cDNA synthesis by reverse transcription using a ProtoScript AMV First Strand cDNA synthesis kit (New England BioLabs) with 1 µg of the isolated total RNA. Quantitative PCR was performed using primers specific for *tnaA* and *rpoB* genes (control). Known quantities of *tnaA* DNA were used as standards. Mann-Whitney U test showed a significant difference (*P > *0.001) in the *tnaA* transcript levels detected in both E. coli strains cultured in the presence and absence of the C. difficile but no significant difference (*P *= 0.401) between the two E. coli strains. Samples of the RNA preparation processed without the reverse transcription step uniformly yielded no detectable SYBR green signal. Error bars represent the standard deviations from three independent experiments. (B) Effect of the C. difficile Agr1 quorum signaling system on indole production in E. coli. (I) E. coli *H10407* cells were cocultured with and without C. difficile wild-type R20291, *agr1* mutant (*agr1*Mut), and the complemented *agr1* mutant (Comp*agr1*Mut) for 24 h anaerobically. (II) E. coli H10407 cells were cocultured with and without purified Agr1 quorum signaling autoinducing peptide (TI signal) for 6 h aerobically at 37°C. The culture supernatants were tested for indole using the hydroxylamine indole assay (15). (C) Effect of indole on growth of anaerobes. The anaerobes were cultured anaerobically for 24 h at 37°C in the presence of different indole amounts (0 to 6 mM). Concentration of indole that completely inhibited bacterial growth was recorded. Data shown represent three independent replicates.

To examine how C. difficile induces indole production, the expression of the tryptophanase gene (*tnaA*) in the E. coli strains was measured by quantitative real-time PCR. E. coli strains exposed to C. difficile stationary-phase supernatant expressed significantly (*P *= 0.0025) higher *tnaA* levels (>4-fold) than when cultured alone (Fig. 4A). The increased expression of *tnaA* in the presence of the stationary-phase culture supernatant suggested that C. difficile likely induces indole at the level of transcription.

Since C. difficile inhabits the colon with other gut microbiota, we investigated whether it can also induce indole in other non-E. coli indole producers that coinhabit the colon. Remarkably, several indole-positive facultative and obligate anaerobes cocultured with C. difficile cells produced elevated indole levels (see Fig. S2A). In contrast, the amount of indole produced by E. coli cells cocultured with many of these anaerobes (as control) was not significantly different (*P *= 0.4240) from the amount produced by the E. coli cells alone (Fig. S2B). These results indicated that the indole-inducing phenomenon is associated with C. difficile strains and underscores the role of this pathogen in controlling the physiology of other gut bacteria.

10.1128/mSystems.00346-18.2 FIG S2 (A) C. difficile induces indole production in other indole-producing anaerobes. C. difficile was cocultured with other obligate and facultative anaerobes for 24 h at 37°C. (B) Other anaerobes cannot induce indole in E. coli strains. E. coli strains H10407 and 25922 were cultured anaerobically with other obligate and facultative anaerobes for 24 h at 37°C. Indole concentration in the respective supernatants was determined in triplicates using the hydroxylamine indole assay. Representative data from three independent experiments are shown. Download FIG S2, TIF file, 0.2 MB. Copyright © 2019 Darkoh et al. 2019 Darkoh et al. This content is distributed under the terms of the Creative Commons Attribution 4.0 International license.

### The accessory gene regulator 1 quorum signaling system is involved in induction of indole production.

We previously demonstrated that the Agr1 quorum signaling system regulates toxins A and B production in C. difficile (10, 11). To investigate whether the Agr1 system may also be involved in indole induction, a C. difficile R20291 *agr1* mutant unable to produce the quorum signal or the purified Agr1 quorum signaling peptide was tested in E. coli cells. The amount of indole produced by the E. coli H10407 strain in coculture with the R20291 *agr1* mutant was significantly (*P < *0.0010) lower than the amount produced when cocultured with the wild-type C. difficile R20291 strain (Fig. 4B-I). However, E. coli H10407 cells cocultured with the complemented *agr1* mutant produced more indole than the E. coli cells alone or in coculture with the *agr1* mutant. Furthermore, the autoinducing quorum signaling peptide from Agr1 (TI signal) purified from a culture supernatant induced indole production in the E. coli H10407 cells in a dose-dependent manner (Fig. 4B-II). Moreover, purified TI signal from the CDI-positive stools (Fig. 1) also induced indole production. These results provided evidence demonstrating that induction of indole in the indole-producing bacteria by C. difficile is associated with the Agr1 quorum signaling system.

### Indole inhibits bacterial growth.

Indole exhibits the characteristics of an ionophore and may interact with bacterial cell walls and create redox imbalances that could influence cell viability. To investigate whether indole could affect growth, the MICs of indole against various gastrointestinal bacteria were determined. The results indicated that different gut bacteria have different tolerances for indole and that the pathogenic anaerobes tended to have a higher tolerance for indole than the nonpathogenic ones (Fig. 4C). Furthermore, the anaerobes that are known to confer human-derived benefits in the colon, such as *Bacteroides* species, tended to have a lower indole tolerance. Interestingly, the indole concentrations observed in the stools of C. difficile patients were higher than the MICs of most of the bacteria tested, suggesting that the growth of these bacteria during CDI may be affected. Together, these results demonstrate that C. difficile induces indole production in other indole-producing bacteria and that the increased indole level may be detrimental to some of the beneficial bacteria conferring colonization resistance in the gut.

## DISCUSSION

The human gut serves as the host to a repertoire of microbial taxa in various abundances and compositions. The diverse composition of the gut microbiota mediates human health by promoting defense against pathogen colonization (1, 2). Accordingly, there is an increasing interest in understanding how the gut microbiota interact among each other. Such interactions might be mutualistic; for example, a metabolite produced by one bacterium could be useful to others or one bacterium might degrade antibiotics to enable others to survive. In contrast, other interactions could lead to competition for limiting resources such as nutrients (3), adhesion sites (4, 5), and other useful metabolites (6, 7). One of the important microbially derived metabolites produced by certain bacteria is indole, a potent antimicrobial antioxidant. Here, we demonstrate that C. difficile (a non-indole producer) induces indole production in other indole-producing bacteria. C. difficile induced increasing expression of the *tnaA* gene required to metabolize tryptophan into indole in E. coli. Other indole-producing bacteria also produced more indole when cocultured with C. difficile. Remarkably, a C. difficile mutant of the accessory gene regulator 1 (Agr1) quorum signaling system that is unable to produce the quorum signaling autoinducing peptide did not induce indole. However, the mutant was able to induce indole when Agr1 quorum signaling was restored by complementation, suggesting that the Agr1 system might play a role in the C. difficile-mediated induction of indole production.

Moreover, stools from CDI patients showed higher indole levels than non-CDI diarrhea stools, demonstrating that this phenomenon occurs during infection. In addition, the amount of indole detected in the stools of CDI patients was higher than the indole MICs of most of the bacteria colonizing the gut. We propose that C. difficile induces indole during infection, which inhibits the beneficial gut-protective and indole-sensitive microbiota, and this promotes the propagation of indole-tolerant bacteria, leading to reduced colonic microbial diversity and dysbiosis that sustain CDI. To our knowledge, the activation of indole production by a non-indole-producing bacterium such as C. difficile through a quorum signaling mechanism has not been reported in the literature and uncovers a novel interaction among the gut microbiota. Our research is ongoing to further understand the nature of this interaction and how it impacts the host and disease clearance.

**FIG 5:**
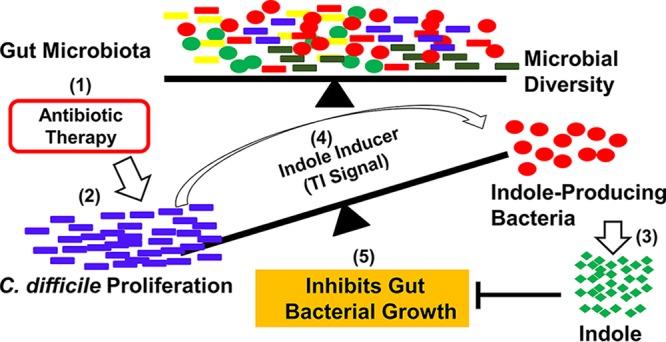
A proposed model for interaction of the gut microbiota and indole production during C. difficile infection. (1) Antibiotic therapy alters the balance in composition and diversity of the gut microbiota leading to dysbiosis. (2) This allows C. difficile to proliferate in the colon. (3) Antibiotic therapy also selects for indole-producing bacteria such as *Proteobacteria* that persist in the gut. During dysbiosis from diet or antibiotic use, *Proteobacteria* overgrow from levels of approximately 5% normally to up to 50% of the microbiota (40, 41). (4) Subsequently, the burgeoning number of C. difficile cells leads to production of the Agr1 quorum signal (TI signal), which in turn activates indole production. (5) The increased indole concentration limits the growth of the beneficial indole-sensitive bacteria in the colon, further disrupting the colonization resistance and allowing C. difficile to persist.

We think that antibiotic therapy alters the homeostasis in composition and diversity of the gut microbiota leading to dysbiosis. This allows C. difficile to proliferate in the colon, but the antibiotic therapy also selects for resistant indole-producing bacteria such as *Proteobacteria* that persist in the gut. During dysbiosis from diet or antibiotic use, *Proteobacteria* overgrow from levels of approximately 5% normally to up to 50% of the microbiota (40, 41). Subsequently, the burgeoning number of C. difficile cells increases the production and level of the Agr1 quorum signal, which in turn activates indole production in the surrounding bacteria capable of producing indole. The increasing indole levels limit the growth of beneficial indole-sensitive bacteria in the colon, such as *Bacteroides* and *Edwardsiella*, further altering colonization resistance with attendant dysbiosis that enables C. difficile to persist (Fig. 5). Altogether, these results may explain a mechanism of C. difficile persistence during infection and provide insight into the complex interactions and chemical warfare among the gut microbiota.

## MATERIALS AND METHODS

### Reagents, bacterial strains, and growth conditions.

All of the bacteria stocks used in the study, including E. coli H10407 (ATCC 35401) and 25922 (ATCC 25922) were either purchased from the American Type Culture Collection (Manassas, VA) or frozen stocks of clinical isolates stored in our laboratory. Brain heart infusion (BHI) medium was purchased from Becton Dickinson (Cockeysville, MD). Hydroxylamine-HCl and l-tryptophan were purchased from Sigma-Aldrich (St. Louis, MO). Anaerobic growth conditions were maintained at an atmosphere of 10% H_2_, 5% CO_2_, and 85% N_2_ in a Bactron-600 anaerobic chamber (Sheldon Manufacturing, Inc., Cornelius, OR).

### Ethics statement.

The deidentified clinical stool samples used in this study were obtained from an ongoing study approved by the Institutional Review Boards (IRBs) of The University of Texas Health Science Center at Houston and a major hospital at the Texas Medical Center (Houston, TX). All the participating patients or their legal guardians provided written informed consent before their stool samples were collected.

### C. difficile confirmation in stools.

Upon collection from patients, the samples were transported on ice packs to the laboratory, aliquoted, and stored in −80°C until analyzed. The IRB approval stipulated that patient stool samples must be deidentified prior to analysis in our laboratory. As a result, clinical data associated with the stool samples were excluded from the study.

All the stool samples were initially tested by either tissue culture cytotoxicity assay or real-time PCR and classified as either C. difficile positive (CDI positive) or C. difficile negative (CDI negative) by the medical microbiology laboratory at the hospital. Upon arrival in our laboratory, the stools were further confirmed by toxigenic culture using the C. difficile plate assay and PCR (42–44).

### Purification of indole from patient stools.

Two pools of patient stools (1 g each) consisting of 20 CDI-positive samples in one group and 20 CDI-negative samples in another group were resuspended in 100 ml of phosphate-buffered saline (PBS). The suspension was centrifuged for 20 min at 15,000 × *g* to remove debris, and the supernatant was carefully decanted. The pellet was resuspended two more times in 100 ml of PBS until approximately 250 ml of supernatant was obtained. The supernatant fluid was mixed with 70% ice-cold acetone and incubated overnight at −20°C to allow precipitation of small molecules. The precipitate was centrifuged at 15,000 × *g* for 20 min, and the pellet was resuspended in 50 ml of PBS and filtered through a centrifugal filter with 3-kDa-cutoff membrane (Sigma-Aldrich, St. Louis, MO) to eliminate large-molecular-weight compounds. The filtrate was concentrated in a SpeedVac (Thermo Fisher Scientific, Waltham, MA) and purified by preparative high-performance liquid chromatography (HPLC) using an Econosil C_18_ column (250 mm by 10 mm; Alltech) with a Shimadzu Prominence HPLC system (Shimadzu Scientific Instruments, Columbia, MD). Each purification run was performed by injecting 1 ml of the sample and washing with buffer A (97.5% [vol/vol] acetic acid-H_2_O; pH 3.8) to eliminate unbound materials. The bound compounds were fractionated by gradient elution using buffer B (80%:20% [vol/vol] acetonitrile/H_2_O). The fractions of interest were pooled and further purified by analytical HPLC with a smaller Econosil C_18_ column (4.6 mm by 150 mm) using the buffer conditions described above. To identify the unique fraction in the CDI-positive samples, the purified material was sent to Moore Analytical (Houston, TX) for mass spectrometry and Fourier transform infrared spectroscopy analysis.

### Indole assay.

Indole concentrations in the stools were determined using the hydroxylamine indole assay (15). Briefly, 250 mg of stool was suspended in 750 µl of 70% ethanol, vortexed for 30 s, and incubated in a 70°C water bath for 10 min. The samples were vortexed again for 30 s and centrifuged at 14,000 × *g* for 20 min at 40°C, and the supernatants were carefully pipetted. For the indole test, 100 µl of the supernatant was added in triplicates to a Costar 96-well plate (Corning, NY) containing 25 µl of NaOH (5.3 M) and 50 µl of 0.3 M hydroxylamine hydrochloride (NH_2_OH-HCl) and incubated for 15 min at room temperature. Following the incubation period, 125 µl of H_2_SO_4_ (2.7 M) was added, thoroughly mixed, and incubated at room temperature for 2 to 30 min. Absorbance at a 530-nm wavelength was measured using a Spectramax I3 spectrophotometer (Molecular Devices, Sunnyvale, CA). The concentration of indole in the sample was determined using a standard curve obtained from known indole concentrations.

### Measurement of indole in bacterial cultures.

For the coculture experiments, overnight cultures were prepared from single colonies of each bacterium tested. The optical density at 600 nm (OD_600_) of each culture was adjusted to 0.5, and 50 µl of the culture was inoculated into 4.9 ml of BHI broth supplemented with 5 mM l-tryptophan. Then, 50 µl of C. difficile strains 630 or R20291 overnight culture (adjusted to an OD_600_ of 0.5) was added to a final volume of 5 ml under anaerobic conditions and incubated at 37°C for 20 h. For the indole test, 1 ml of the resulting coculture was centrifuged at 14,000 × *g* for 15 min, and 100 µl of the supernatant was tested in triplicates using the hydroxylamine-based indole assay described above (15).

To test for the effect of C. difficile culture supernatant on indole production, culture supernatant fluids from C. difficile strains 630 and R20291 were collected at mid-log phase (6 h), early stationary phase (10 h), and late stationary phase (24 h). The supernatants were centrifuged at 14,000 × *g*, filtered using a 0.2-μm filter, and stored at −20°C until analyzed. The tester indole-producing bacteria (30 µl) at an OD_600_ of 0.5 were inoculated in fresh BHI broth supplemented with 5 mM l-tryptophan and different amounts (0%, 25%, 50%, and 75%) of the cell-free 0.2-μm-filtered C. difficile culture supernatant fluid in a final volume of 3 ml. The culture was incubated either anaerobically or aerobically at 37°C with shaking at 250 rpm for 4 to 6 h, and the supernatant fluid was tested for indole, as described above. Both boiled (culture boiled for 10 min) and unboiled supernatants were also tested.

To test for effect of the Agr system on indole production, a C. difficile R20291 mutant deficient in Agr1 quorum signaling (*agr1*Mut), its complemented mutant (Comp*agr1*Mut), and the wild type were used. Also, partially purified Agr1 autoinducing quorum signaling peptide (TI signal) was tested. The methods used to generate the *agr1* mutant and purification of the TI signal were previously reported (10, 11, 45). For the assay, an overnight culture of the E. coli H10407 strain (30 µl) at an OD_600_ of 0.5 was cocultured with the wild-type R20291, *agr1*Mut, or Comp*agr1*Mut (30 µl each at an OD_600_ of 0.5) in fresh BHI broth in a total volume of 3 ml. To maintain the complemented plasmid, an overnight culture of the Comp*agr1*Mut strain was made in BHI broth containing 50 µg/ml chloramphenicol and washed twice with fresh BHI broth before adding to the E. coli H10407 cells. The cocultures were incubated anaerobically for 24 h, and the culture supernatants were tested for indole.

To test for the effect of TI signal, E. coli H10407 cells (30 µl) were added to fresh BHI broth containing 5 mM l-tryptophan and different amounts of the partially purified TI signal (1, 10, and 20 µg/ml) in a final volume of 3 ml. The culture was incubated aerobically for 6 h at 37°C, and the supernatant was tested for indole. The total protein concentration of the culture was determined using the Bradford assay (46).

### Analysis of *tnaA* transcription.

Overnight cultures of E. coli strains H10407 and 25922 at an OD_600_ of 0.5 were diluted 1:100 in fresh BHI medium containing 25% cell-free 0.2-μm-filtered C. difficile 24-h culture supernatant fluid and 5 mM l-tryptophan in a final culture volume of 10 ml. As control, the E. coli strains were cultured in fresh BHI medium only or autoclaved 25% E. coli 24-h culture supernatant. The culture was incubated anaerobically for 6 h at 37°C. Total RNA was isolated using an RNeasy kit (Qiagen) according to the manufacturer’s instructions. The RNA (1 μg) was converted into cDNA by reverse transcription using a ProtoScript AMV First Strand cDNA synthesis kit (New England BioLabs, Ipswich, MA) according to the manufacturer’s instructions. The cDNA was diluted 1:1 with RNase-free water, and the relative expression level of *tnaA* transcripts was determined using SYBR green JumpStart *Taq* Ready mix (Sigma-Aldrich) containing 500 nM each primer in a final volume of 20 μl. A primer pair specific for the *tnaA* gene (TNA-F, 5′-ACCAGAGCCAAACCGATTGA-3′; TNA-R, 5′-AGCGCGAGTGGTACGTTTTA-3′) was used. Known quantities of *tnaA* DNA were used as the standard. Comparative threshold cycle (*C_T_*) analysis was performed, and the absolute quantitative method was used to calculate the level of *tnaA* transcripts based on the mean expression from three biological replicates. The *rpoB* gene was used as an internal control. To check for DNA contamination, samples of the RNA preparation were processed without the reverse transcription step and yielded no detectable SYBR green signal.

### Determining indole MIC.

The minimum amount of indole required to inhibit the growth of various facultative and obligate anaerobes was determined using the broth dilution method (47–51). Briefly, an overnight culture of the tested bacterium at an OD_600_ of 0.5 was diluted 1:100 in BHI broth (total volume of 300 µl) containing different concentrations of indole from 0 to 6 mM in a sterile 96-well plate. The culture was incubated for 24 h anaerobically at 37°C, and the OD_600_ was measured to determine the indole concentration that completely inhibited bacterial growth.

### Statistical analysis.

Data were analyzed using R software (R Foundation for Statistical Computing, Vienna, Austria) and GraphPad Prism (GraphPad Software, San Diego, CA). To measure the difference in indole levels in clinical stools, the Wilcoxon rank sum test (Mann-Whitney U test) was used. A two-sample *t* test was used to assess the significance between indole induction in indole-producing bacteria with C. difficile coculture and the supernatant. A one-way analysis of variance (ANOVA) was used to test the significance of the dose-dependent indole induction. Statistical significance was defined as a *P* value of <0.05.
