# Supplementary figures and images for: Clostridium difficile Modulates the Gut Microbiota by Inducing the Production of Indole, an Interkingdom Signaling and Antimicrobial Molecule
Source: mSystems. 2019 Mar 19;4(2):e00346-18. doi: 10.1128/mSystems.00346-18 (PMC6426650; doi:10.1128/mSystems.00346-18)

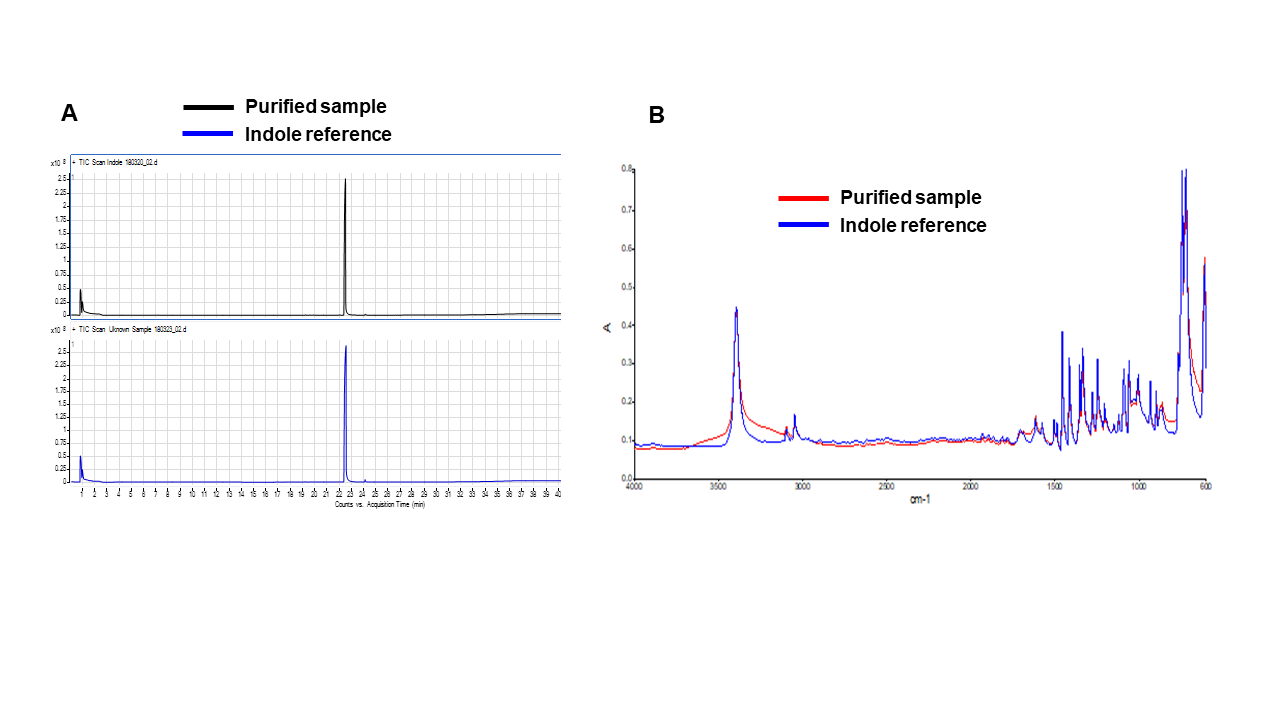

Supplement: FIG S1 [file mSystems.00346-18-sf001.tif]

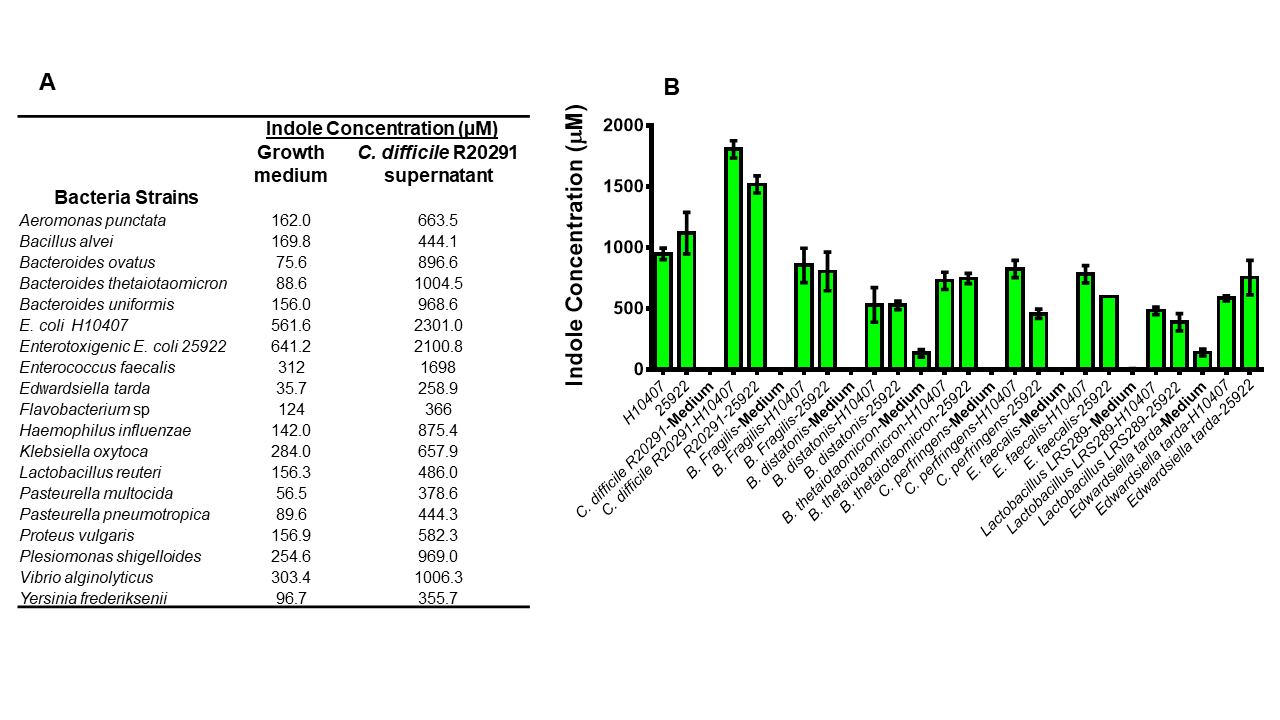

Supplement: FIG S2 [file mSystems.00346-18-sf002.tif]
